# The Role of the Hippo Pathway in Alzheimer's Disease

**DOI:** 10.1002/alz70855_107695

**Published:** 2025-12-24

**Authors:** Maria de Haro, Omar El Fadel, Morgan Catherine Stephens, Kwanghyuk Danny Lee, Olivier Lichtarge, Ismael Al‐Ramahi, Juan Botas

**Affiliations:** ^1^ Baylor College of Medicine, Houston, TX, USA; ^2^ Jan and Dan Duncan Neurological Research Institute, Houston, TX, USA; ^3^ Jan and Dan Duncan Neurological Research Institute, Texas Children's Hospital, Houston, TX, USA; ^4^ Center for Alzheimer's and Neurodegenerative Diseases ‐ BCM, Houston, TX, USA

## Abstract

**Background:**

Genomic and transcriptomic analyses have identified molecular pathways dysregulated in Alzheimer's disease (AD). However, their roles in disease remain largely unknown due to limited in vivo validation. YAP1, a downstream effector of the Hippo pathway, has been shown to be dysregulated in AD and sequestered into amyloid aggregates. Additionally, studies have shown that suppressing Hippo kinase activity improves outcomes in AD animal models (PMIDs: 31980612, 35525373, 38760516). This study explores the interaction between the Hippo pathway and Tau, examining how its dysregulation contributes to AD pathogenesis and providing new mechanistic insights into the therapeutic potential of targeting the Hippo pathway.

**Method:**

We analyzed brain transcriptomes from Alzheimer's disease (AD) patients (bulk and single‐nucleus) and AD mouse models. Additionally, whole‐exome sequencing data from ADSP revealed differential mutational burdens in Hippo pathway genes between AD cases and controls. To validate and characterize the interaction between the Hippo pathway and Tau, we employed behavioral, biochemical, and histopathological assays in vivo and in human NPCs.

**Result:**

Genomic analyses of AD suggest an increased mutational burden in Hippo pathway genes compared to controls, while transcriptomic data indicate dysregulation consistent with pathway activation. These in silico findings were validated in vivo. We demonstrate that Hippo pathway modulation can suppress Tau‐mediated neuronal dysfunction in Drosophila. Additionally, knockdown of several Hippo pathway genes reduces Tau protein levels in both Tauopathy fly and human NPC models. Furthermore, Tau and YAP1 co‐immunoprecipitate in human NPCs and brains from aged P301S mice.

**Conclusion:**

Our findings reveal aberrant Hippo pathway activation and increased mutations in AD. Modulating pathway components reduces Tau toxicity and lowers Tau levels, highlighting the Hippo pathway as a potential diagnostic and therapeutic target.

